# Application of High-Throughput Assays to Examine Phospho-Modulation of the Late Steps of Regulated Exocytosis

**DOI:** 10.3390/ht6040017

**Published:** 2017-11-13

**Authors:** Prabhodh S. Abbineni, Jens R. Coorssen

**Affiliations:** 1 Department of Molecular Physiology, and the WSU Molecular Medicine Research Group, School of Medicine, Western Sydney University, Campbelltown, NSW 2560, Australia; pabbinen@umich.edu; 2 Faculty of Applied Health Sciences and Faculty of Mathematics and Science, Brock University, St. Catharines, ON L2S 3A1, Canada

**Keywords:** phosphorylation, docking, priming, membrane fusion, calcium sensitivity, lipids, secretory vesicles

## Abstract

Regulated exocytosis enables a range of physiological functions including neurotransmission, and the late steps (i.e., docking, priming and Ca^2+^-triggered membrane fusion) are modulated by a highly conserved set of proteins and lipids. Many of the molecular components and biochemical interactions required have been identified; the precise mechanistic steps they modulate and the biochemical interactions that need to occur across steps are still the subject of intense investigation. Particularly, although the involvement of phosphorylation in modulating exocytosis has been intensively investigated over the past three decades, it is unclear which phosphorylation events are a conserved part of the fundamental fusion mechanism and/or serve as part of the physiological fusion machine (e.g., to modulate Ca^2+^ sensitivity). Here, the homotypic fusion of cortical vesicles was monitored by utilizing new high-throughput, cost-effective assays to assess the influence of 17 small molecule phospho-modulators on docking/priming, Ca^2+^ sensitivity and membrane fusion. Specific phosphatases and casein kinase 2 are implicated in modulating the Ca^2+^ sensitivity of fusion, whereas sphingosine kinase is implicated in modulating the ability of vesicles to fuse. These results indicate the presence of multiple kinases and phosphatases on the vesicles and critical phosphorylation sites on vesicle membrane proteins and lipids that directly influence late steps of regulated exocytosis.

## 1. Introduction

Eukaryotic cells release select compounds via regulated exocytosis in order to enable a range of physiological functions (e.g., neurotransmission, immunological reactions, fertilisation, wound healing, autophagy, and so forth). This regulated exocytotic pathway is often categorised into distinct functional stages including: (i) targeted trafficking of secretory vesicles to the plasma membrane; (ii) tethering at the plasma membrane; (iii) full docking; (iv) priming (which may also include pre-docking molecular changes); (v) triggering; (vi) membrane fusion; (vii) fusion pore regulation and/or expansion; and (viii) content release. Molecular components that regulate exocytosis can be broadly categorised as either modulating the efficiency of release (i.e., the physiological fusion machine (PFM) influencing parameters such as Ca^2+^ sensitivity and the rate of fusion) or as components of the fundamental fusion mechanism (FFM), which directly enables the process of membrane merger leading to the opening of the fusion pore and the release of intracellular compounds [1,2,3,4,5]. Several proteins and lipids, including soluble *N*-ethylmaleimide-sensitive factor attachment proteins (SNAREs), cholesterol, poly-phosphatidylinositols and anionic lipids, are known to be involved in enabling and regulating different stages of the exocytotic pathway and have been confirmed as critical elements of the PFM, with a few now also confirmed as components of the FFM in native systems [1,6,7,8,9,10,11].

The activity of proteins and lipids involved in exocytosis can be modulated by kinases and phosphatases, which are capable of regulating inter-protein, inter-lipid and protein­–lipid interactions or of triggering conformational changes necessary for a specific stage of the exocytotic pathway. For example, numerous studies have found that phorbol 12-myristate 13-acetate (PMA), which activates protein kinase C (PKC), also potentiates exocytotic release in a range of cell types [12,13,14]; phosphorylation of synaptosomal-associated protein 25 (SNAP-25) by PKC has been suggested to account for this by increasing the size of the readily releasable pool of vesicles in chromaffin cells [15]. However, overexpression of a SNAP-25 mutant that cannot undergo phosphorylation does not inhibit the enhancing effect of PMA in hippocampal neurons. Interpretation has been further confounded in this case by the finding that PMA also interacts with Munc13 and guanosine triphosphate (GTP)-binding proteins [16,17]. Furthermore, activation of several kinases and phosphatases has been reported to have seemingly opposing functional effects; tyrosine kinase activation has been shown to potentiate release in β cells [18], but to act as a negative regulator, inhibiting transmitter release from neuronal cells [19]. Likewise, inhibiting calcineurin (a Ca^2+^/calmodulin-dependent protein phosphatase) has been found to potentiate release from β cells and rat cerebral cortex synaptosomes [20,21], but to inhibit the release of pepsinogen from permeabilised chief cells and to reduce secretion from the rapidly releasable pool of vesicles in hippocampal neurons [22,23].

Given these contrasting results, it has clearly been difficult to definitively correlate phosphorylation-related events with specific stages of the regulated exocytotic pathway. To some degree, this has likely been complicated by alternate and/or off-target effects of ‘selective’ chemical inhibitors and activators, as well as the model systems and assessment techniques (i.e., intact, permeabilised or patch clamped cells). As such, it remains to be defined which phosphorylation events enable or modulate particular stages of the exocytotic pathway and thus to discriminate between conserved features of the fundamental exocytotic machinery versus those that may have been selectively optimised over evolution to ‘tune’ specific physiological responses.

As cortical vesicles (CV) isolated from sea urchin oocytes are locked in a fully primed, release-ready state, they are an ideal, well-established model with which to assess the role(s) of highly conserved proteins and lipids in the Ca^2+^-triggered stages of exocytosis [2,3,5,7,8,9,10,24,25,26,27,28]. The sea urchin genome encodes for all of the kinase classes present in vertebrates [29,30], and CV membrane-associated proteins and lipids are known to be sufficient for docking, Ca^2+^ sensing and membrane fusion via a mechanistic pathway that is by all accounts identical to vesicle–plasma membrane fusion [7,24,26,31,32]. Therefore, if a (de)phosphorylation event is required to enable or modulate the Ca^2+^ triggered steps of regulated exocytosis, the kinase(s) and phosphatase(s) responsible likely target components of the secretory vesicle membrane and are perhaps components themselves. Kinases present in the cytosol or plasma membrane could also be involved in modulating the PFM, but their influence would not be detected in this study (although the assay could clearly be modified by also adding isolated cytosol to address this). It would however seem reasonable to expect that any components critical to the late mechanistic steps of exocytosis would be preferentially localized to the CV and/or to vesicle–plasma membrane contact sites. Here, we have used CV and minimised docking and membrane fusion assays to screen a selection of well-characterised small molecule (including peptide) modulators of phosphorylation. Two sub-maximal doses of [Ca^2+^]_free_ were used to trigger a fraction of CV to fuse, and this minimised assay enabled us to rapidly screen for effects on Ca^2+^ sensitivity. This approach is fundamentally similar to one previously utilised by Vogel et al. [33] to identify membrane active drugs; however, that study focussed on a generic testing of drugs by utilising cell surface complexes (sheets of plasma membrane with endogenously docked CV), which do not enable discrimination of drug effects on docking/priming and membrane fusion, which was made possible here by monitoring homotypic CV fusion using minimised Ca^2+^ activity assays of docking and fusion. The resulting high-throughput, microplate reader-based assays enabled massively parallel screening not possible with the full activity assay formats previously employed. The results implicate specific kinases and phosphatases in enabling and modulating the fundamental mechanisms underlying the late Ca^2+^ triggered steps of regulated exocytosis.

## 2. Materials and Methods

### 2.1. Materials

*Heliocidaris tuberculate* (red sea urchins) were collected under government licence from Shellharbour and La Perouse (NSW, Australia) and stored in aquaria at 10 °C in the aquatic facility of Western Sydney University. Phorbol 12-myristate 13-acetate (PMA) and staurosporine were purchased from Sigma Aldrich (St. Louis, MO, USA); okadaic acid was from Calbiochem (San Diego, CA, USA); and all other small molecule phosphorylation inhibitors were from Tocris (Ellisville, MO, USA). Double glass-distilled water (ddH_2_O) was used throughout. All fusion assays were performed using the POLARstar Omega plate reader (BMG Labtech, Offenburg, Germany).

### 2.2. Isolation of Cortical Vesicles and Drug Treatments

Cortical vesicles were isolated from urchin oocytes as previously described, with some modifications [5,24,27]. Cell surface complexes (CSC), large plasma membrane fragments with endogenous docked vesicles, were obtained by homogenising oocytes on ice and isolated by centrifugation at 700× *g* for 5 min. Cell surface complexes were then washed free of cytosolic components using ice cold intracellular medium (IM; 210 mM glutamate (free acid), 0.5 M glycine, 10 mM NaCl, 10 mM piperazine-*N*,*N*′-bis(2-ethanesulfonic acid (PIPES), 1.1 mM MgCl_2_ and 5 mM EGTA, pH 6.7) and then incubated in PKME buffer (50 mM PIPES (free acid), 425 mM KCl, 10 mM MgCl_2_, 5 mM ethylene glycol-bis(β-aminoethyl ether)-*N*,*N*,*N′*,*N′*-tetraacetic acid (EGTA), pH 8.0 (KOH)) for 30 min to disassociate CV from the plasma membrane. Cortical vesicles were than isolated from plasma membrane fragments by centrifugation at 700× *g* for 5 min, followed by centrifugation at 2000× *g* for 20 min to obtain a high purity pellet of CV. All functional assays and incubations with small-molecule activators and inhibitors were carried out in baseline intracellular medium (BIM; 210 mM glutamate (free acid), 0.5 M glycine, 10 mM NaCl, 10 mM PIPES (free acid), 0.05 mM CaCl_2_, 1 mM MgCl_2_, 1 mM EGTA) supplemented with 2.5 mM ATP, protease inhibitors and 2 mM dithiothreitol (DTT) [8,9,26,27]. Cortical vesicles treatments and endpoint and settle fusion assays were performed as previously described [7,9,10,27,34]. Briefly, CV were treated with the indicated concentrations of drugs for 20 min at 25 °C at an optical density (OD_405_) of 1.0 and immediately diluted with ice-cold BIM. α-naphthyl phosphate (α-NP) was delivered from a 500 mM stock prepared in ddH_2_O, and all other small molecules were delivered from concentrated stocks in dimethyl sulfoxide (DMSO); the final solvent concentration was ≤1%, and although it was confirmed to have no effect on the fusion assays [35], this vehicle was nonetheless added to all controls. Drug concentrations utilised in this study were identified in the literature using PubMed, and in many cases, the compounds used here have been used to assess the exocytotic mechanism in several cell types; see Table 1 for list of drugs, their molecular targets, concentration used and references. Cortical Vesicles density was adjusted to OD_405_ of 0.3–0.4 for functional assays. In the endpoint assay, CV were dispensed into multi-well plates and centrifuged at 1000× *g* for 10 min to bring them into contact, and Ca^2+^ was subsequently added to trigger fusion. In the ‘settle’ assay (which allows vesicles to come into contact utilising endogenous docking machinery), CV were left for 1 h at room temperature (RT) to make inter-membrane attachments before the addition of Ca^2+^.

### 2.3. High-Throughput Methodology

Sub-maximal doses of [Ca^2+^]_free_ trigger a fraction of CV to fuse, and CV fusion vs. [Ca^2+^]_free_ is best described by a sigmoidal Ca^2+^ activity curve [24,36]. Changes in CV fusion triggered by sub-maximal doses of [Ca^2+^]_free_ are indicative of changes to the Ca^2+^ sensitivity of the mechanism(s), while alterations in the response to saturating doses of [Ca^2+^]_free_ (~1 mM) indicate changes to the capacity for vesicles to fuse (i.e., the ability to fuse). In this study, minimised versions of standard endpoint and settle Ca^2+^ activity assays were used to enable high-throughput screening of all the test compounds. Two sub-maximal doses of [Ca^2+^]_free_ (38.8 ± 1.7 µM and 69.8 ± 4.8 µM [Ca^2+^]_free_) were used to trigger CV fusion and were designated as ‘low’ and ‘medium’ Ca^2+^, respectively, in the endpoint assay; 60.7 ± 1.5 µM [Ca^2+^]_free_ and 91.2 ± 4.3 µM [Ca^2+^]_free_ were designated as low and medium Ca^2+^, respectively, in the settle assay. In both assay formats, responses to 1 µM and ≥1 mM [Ca^2+^]_free_ were defined as 0% and 100% fusion, respectively; as is standard for such Ca^2+^ activity assays, ≥1 mM [Ca^2+^]_free_ was thus defined as ‘high’ Ca^2+^. Thus, here, monitoring changes in the range of [Ca^2+^]_free_ corresponding to the rising phase of the standard sigmoidal Ca^2+^ activity curves enabled us to carry out broadly parallel assays with high throughput and thus scan and identify small molecules that altered the Ca^2+^ sensitivity of fusion. Sigmoidal curves were plotted using responses to low and medium doses of [Ca^2+^]_free_ to estimate the transition phase of the sigmoid curve in order to obtain an initial estimate of the half maximum effective concentration (EC_50_). Treatments that were found to significantly alter CV fusion responses to both the low and medium doses of [Ca^2+^]_free_ in the endpoint assay were further assessed using a range of [Ca^2+^]_free_ in order to better define the EC_50_ from full Ca^2+^ activity curves. In all assays, [Ca^2+^]_free_ was measured in parallel mock solutions using a Ca^2+^-sensitive electrode (EDT directION, Dover, UK), calibrated using CALBUF Ca^2+^ standards (World Precision Instruments, Sarasota, FL, USA) [24,25]. All compounds were tested using material from a minimum of three different biological preparations, and each experiment was carried out using three technical replicates. Drug treatments were varied across different experiments. All results are presented as the mean ± SEM. A Shapiro–Wilk test was performed to confirm that the data are normally distributed. The two-tailed Student’s *t*-test was used to assess statistical significance, and *p* < 0.05 was considered significant.

## 3. Results

Seventeen small molecule modulators of phosphorylation (Table 1) were screened for their effect on CV fusion in minimised endpoint and settle assay formats with the aim of identifying candidate kinases and phosphatases that enable or modulate the late Ca^2+^-triggered steps of regulated exocytosis. Changes to CV fusion in response to low and medium doses of [Ca^2+^]_free_ in the endpoint assay are indicative of changes in Ca^2+^ sensitivity, whereas changes in the settle assay (i.e., relative to the endpoint assay) are indicative of alterations to attachment/priming factors [8,9,27]. Inhibition of CV fusion in response to saturating [Ca^2+^]_free_ (mM) in the endpoint assays is indicative of alterations to the membrane fusion machinery, as this assay is thought to bypass the need for those endogenous components required for priming and attachment. Small molecules found to alter CV fusion in response to low and medium doses of [Ca^2+^]_free_ in the minimised endpoint assay were tested again using a more detailed standard Ca^2+^ activity assay (CV fusion versus [Ca^2+^]_free_) to best measure EC_50_.

### 3.1. Effects of Protein Kinase Inhibitors

**Figure 1:**
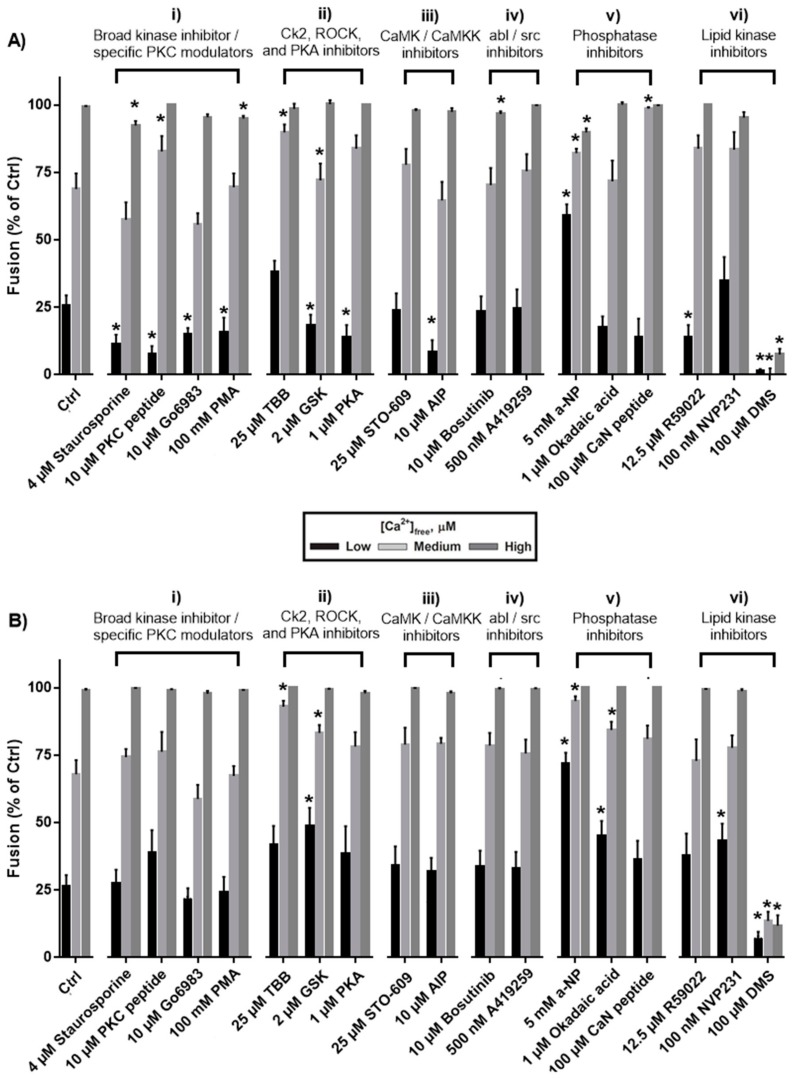
Effects on cortical vesicle–cortical vesicle (CV–CV) fusion in the minimised endpoint (**A**) and settle (**B**) assays broadly inhibiting (i) kinase activities (i.e., with staurosporine), as well as more selectively targeting PKC activity with activators and inhibitors (*n* = 3–5); (ii) inhibiting Ck2, ROCK and PKA (*n* = 3–5); (iii) inhibiting CamK and CamKK (*n* = 3–4); (iv) inhibiting abl/src kinases (*n* = 4–5); (v) broadly inhibiting phosphatase activities (i.e., with α-naphthyl phosphate) and more selectively inhibiting protein phosphatase 1, protein phosphatase 2A and CaN (*n* = 3–5); and (vi) inhibiting diacylglycerol (DAG) kinase, ceramide kinase and sphingosine kinase (*n* = 3–4). * Statistical significance from control (*p* < 0.05). CaMKK: calmodulin-dependent protein kinase kinase.

**Figure 2:**
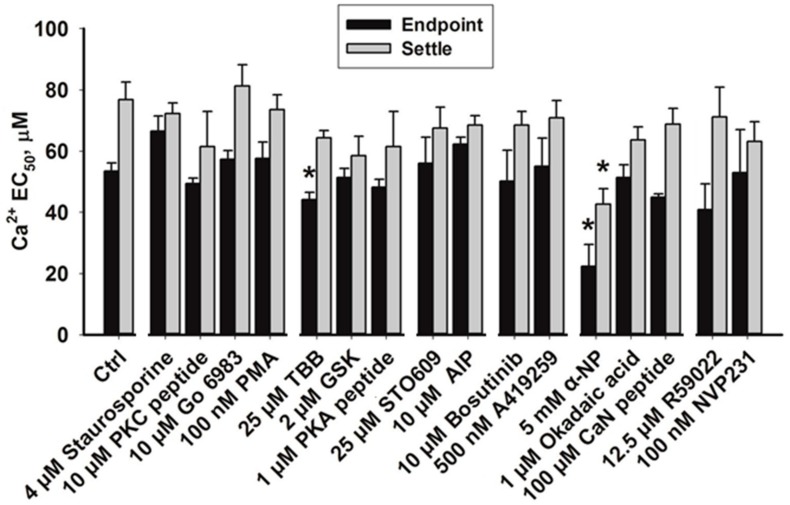
Half maximum effective concentration (EC_50_). estimated using data from minimised endpoint and settle assays. AIP: Autocamtide-2-related inhibitory peptide.

**Figure 3:**
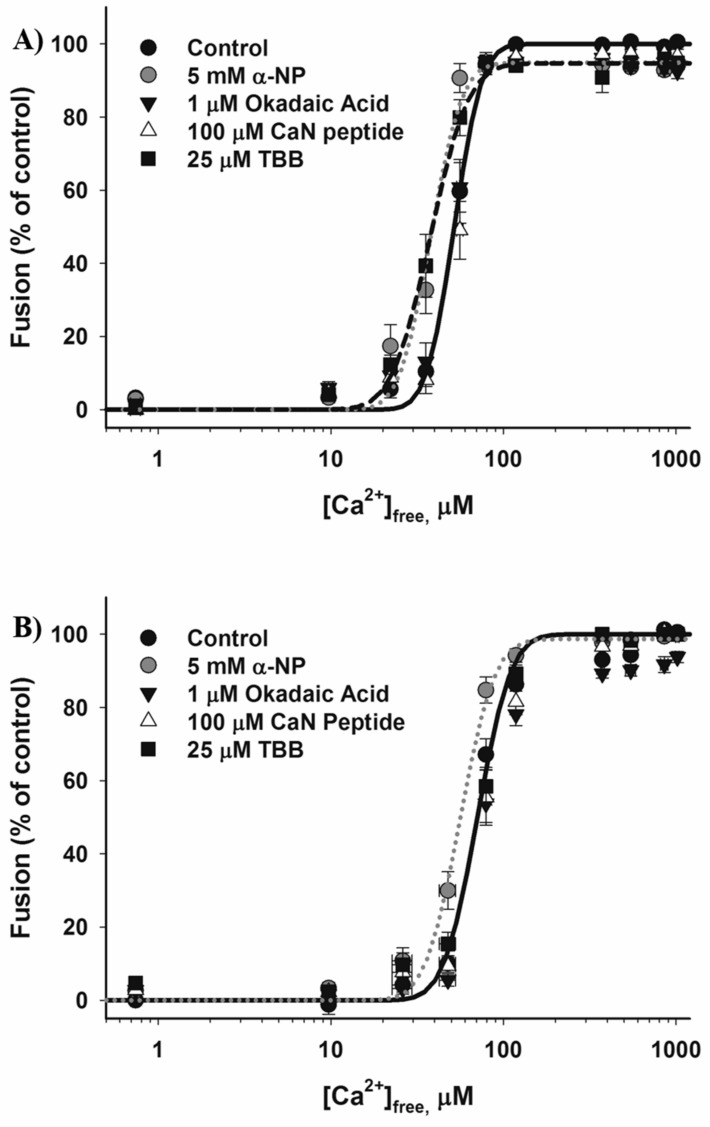
The effects of phosphatase inhibitors and TBB on CV–CV fusion. Full endpoint (**A**) and settle (**B**) Ca^2+^ activity curves (*n* = 3).

We first tested the effect of treating CV with the broad spectrum kinase inhibitor, staurosporine [95]. In the endpoint assay, 4 µM staurosporine caused a reduction in CV fusion in response to low doses of [Ca^2+^]_free_, suppressed the total extent of CV fusion by 8.2% ± 1.6%, but had no effect in the settle assay (Figure 1(Ai,Bi)). Although staurosporine is often utilised as a ‘broad-spectrum’ kinase inhibitor at low micromolar doses, substantially higher concentrations than used here are known to be required to inhibit certain kinases (e.g., >100 µM is required to inhibit casein kinase-1 [96]); however, possible off-target effects involving inhibition of other ATP-binding proteins have been reported, making the effects of higher doses of the drug difficult to interpret [97]. As such, a range of well-characterised, selective small-molecule modulators of phosphorylation were also utilised to test the role(s) of kinase activities in the late steps of regulated exocytosis. Nine of the small molecules tested inhibit protein kinase activities, and of these, only treatment with 4,5,6,7-tetrabromobenzotriazole (TBB), an inhibitor of casein kinase-2, was found to alter CV fusion in response to both low and medium doses of [Ca^2+^]_free_. Treatment with 25 µM TBB promoted the extent of CV fusion in response to low and medium doses of [Ca^2+^]_free_ from control values of 25.6% ± 3.9% and 68.9% ± 5.7% to 38.3% ± 4% and 88.5% ± 2.5%, respectively (Figure 1(Aii)). Standard endpoint Ca^2+^ activity assays confirmed a potentiation of Ca^2+^ sensitivity, with EC_50_ being left-shifted from 52.0 ± 2.5 µM to 33.6 ± 5.7 µM [Ca^2+^]_free_ (Figure 2A and Figure 3A; *p* < 0.05). 4,5,6,7-Tetrabromobenzotriazole also potentiated the extent of CV fusion in the settle assay in response to a medium dose of [Ca^2+^]_free_ without significantly altering the EC_50_ (Figure 3B). The data thus indicate a native role for casein kinase 2 in negatively modulating Ca^2+^ sensitivity.

Treatment of CV with small molecule inhibitors of PKC, PKA and calmodulin-dependent protein kinase II (CaMK II) caused 61.3% ± 10.4% inhibition of fusion in response to low doses of [Ca^2+^]_free_ without significantly affecting fusion in response to medium doses of [Ca^2+^]_free_ (Figure 1(Ai–Aiii)), but had no effect in settle assays (Figure 1(Bi–Biii)). Inhibition of CV fusion in response to low doses of [Ca^2+^]_free_ is indicative of a reduction in Ca^2+^ sensitivity; however, as the inhibition was overcome by medium doses of [Ca^2+^]_free_, it is likely reflective of a significant albeit selective ‘tuning’ of Ca^2+^ sensitivity within a particular concentration range. Thus, the overall EC_50_ was not found to be significantly different relative to controls (Figure 2A,B). Gö 6983, a broad-spectrum PKC inhibitor, supressed the maximal extent of CV fusion by only 4.5% ± 1.1% and did not significantly alter Ca^2+^ sensitivity (Figure 1(Ai)). The PKC activator PMA (100 nM) did not alter Ca^2+^ sensitivity (i.e., did not alter CV fusion in response to low and medium doses of [Ca^2+^]_free_), but suppressed total fusion extent by 5.0% ± 1.0% (Figure 1(Ai)).

The Rho kinase (ROCK) inhibitor, GSK 429286, had no effect on the total extent of CV fusion (Figure 1(Aii)), but selectively potentiated fusion in response to low and medium [Ca^2+^]_free_ in the settle assay only (Figure 1(Bii)); the EC_50_ also appeared slightly left-shifted, but the effect was not significant (Figure 2B). The effects are thus indicative of an upstream role for ROCK in modulating CV docking/priming. Inhibition of abl/src kinases with bosutinib and A419259 did not significantly alter CV fusion in response to low and medium [Ca^2+^]_free_ in the endpoint and settle assays (Figure 1(Aiv,Biv)), though bosutinib caused minor inhibition (3.3% ± 1.0%) of the final extent of fusion.

### 3.2. Effects of Phosphatase Inhibitors

Following treatment with 5 mM α-naphthyl phosphate (α-NP), a broad-spectrum phosphatase inhibitor, CV fusion was potentiated by 60.0% ± 4.7% and 22.4% ± 8.8%, respectively, in response to low and medium doses of [Ca^2+^]_free_ in both the endpoint and settle assays (Figure 1(Av,Bv)). Standard Ca^2+^ activity assays confirmed a potentiation of Ca^2+^ sensitivity, with the control EC_50_ (52.0 ± 2.5 µM [Ca^2+^]_free_) left-shifted to 33.6 ± 5.5 µM [Ca^2+^]_free_ in the endpoint assay and from 82.1 ± 5.7 µM to 56.8 ± 6.9 µM [Ca^2+^]_free_ in the settle assay (Figure 3A,B; *p* < 0.05). The change in EC_50_ is thus comparable in both assay formats indicating that the potentiation of Ca^2+^ sensitivity by α-NP was less likely due to enhanced docking or priming since the endpoint assay is thought to bypass endogenous components essential to these early steps. Treatment with 5 mM α-NP also suppressed the extent of CV fusion in the endpoint assay by 10.1% ± 3.4% (Figure 1(Av)). To narrow the list of possible phosphatases that could be causing the functional changes, okadaic acid, an inhibitor of protein phosphatases 1 and 2A, and calcineurin inhibitory peptide (CaN peptide), an inhibitor of calcineurin (CaN; protein phosphatase 3), were utilised. Treatment with 1 µM okadaic acid increased CV fusion by 41.2% ± 8.6% (*p* < 0.05) only in the settle assay, in response to low and medium [Ca^2+^]_free_ (Figure 1(Bv)); there was no effect on the endpoint assay (Figure 1(Av)). In contrast, exposure to 100 µM CaN peptide selectively and maximally potentiated CV fusion in response to medium doses of [Ca^2+^]_free_ in the endpoint assay (Figure 1(Av)), but had no significant effect in the settle assay (Figure 1(Bv)). Standard Ca^2+^ activity measurements revealed no changes in the EC_50_ following treatment with okadaic acid or CaN peptide (Figure 3A,B).

### 3.3. Effects of Inhibiting Lipid Kinases

Treatment with 12.5 µM R59022, a diacylglycerol kinase inhibitor, inhibited CV fusion in response to low [Ca^2+^]_free_ in the endpoint assay, but had no effect on CV fusion in response to any other dose of [Ca^2+^]_free_ tested in the endpoint and settle assays (Figure 1(Avi,Bvi)) and did not significantly alter EC_50_ (Figure 2A,B). Treatments with NVP231 and dimethylsphingosine (DMS)—inhibitors of ceramide and sphingosine kinases, respectively—were used to assess the role(s) of sphingolipid phosphorylation. Treatment of CV with NVP231 increased fusion in response to a low dose of [Ca^2+^]_free_ in the settle assay (Figure 1(Bvi)), but did not have any effect on responses to low and medium [Ca^2+^]_free_ (Figure 1(Avi)), indicative of a role for ceramide kinase in modulating docking/priming. Treatment of CV with 100 µM DMS had the most potent effects of any of the compounds tested in this study, inhibiting the extent of CV fusion by 92.4% ± 2.1% and 88.2% ± 3.8% (*p* < 0.001) in the endpoint and settle assays, respectively (Figure 1(Avi,Bvi)).

## 4. Discussion

New assay formats enabling high-throughput, parallel screening were used to examine the effect of phospho-modulators on the late stages of regulated exocytosis. Whereas cell surface complexes were previously used to more broadly identify membrane active drugs [33], here we utilised CV, enabling us to target the late steps of the exocytotic mechanism, notably docking/priming, calcium sensitivity and triggered fusion, without the high molecular background of the plasma membrane, which is present in cell surface complexes. We assessed twelve small molecules known to modulate the activity of eleven classes of protein kinases and phosphatases, three small molecules targeting three classes of lipid kinases and two small molecules that act as broad-spectrum kinase and phosphatase inhibitors (Table 1). Of these seventeen compounds, four were found to potentiate and one to markedly inhibit fusion. None of the compounds tested affected CV integrity; changes in optical density only occurred in response to increasing [Ca^2+^]_free_. Utilising both endpoint and settle assays enabled us to distinguish between effects on docking/priming versus membrane fusion, as an additional low-speed centrifugation step in the endpoint assay is thought to bypass the need for endogenous docking/priming machinery. The settle assay cannot distinguish between effects on priming versus docking, and treatments found to selectively alter CV fusion in the settle assay could be indicative of changes to either or both pre-fusion steps.

The sea urchin genome encodes for all of the kinase classes found in vertebrates, and as urchins diverged prior to the advent of whole genome duplications, their genome has a lower degree of redundancy (i.e., in many cases, subfamilies of kinases have only one member) [29,30]. As such, the sea urchin CV model system is ideal for identifying the evolutionarily-conserved components underlying the fundamental Ca^2+^-triggered steps of exocytosis [2,3,7,8,9,24,25,26,27,28,34]. Kinase activity is modulated via allosteric mechanisms by which binding of kinase activators (e.g., Ca^2+^, diacylglycerol, phospholipids) to regulatory domains results in the activation of catalytic sites (containing the ATP binding domain) that bind and phosphorylate the substrate(s). Inhibitors that bind regulatory domains, such as pseudopeptides, tend to show a higher degree of specificity than those that interact with the ATP binding domain (e.g., staurosporine) [95]. This study utilised small molecules that target a range of kinases and phosphatases, with bioinformatic analysis confirming a high degree of conservation between human kinase and phosphatase protein sequences and their urchin progenitors and orthologs (Table 2), particularly at regulatory domains and catalytic sites; this is consistent with previous studies using well-characterised small molecule inhibitors to target urchin enzymes [8,9,27], as well as with the high conservation of other proteins involved in exocytosis [2]. For example, the serine/threonine catalytic domains found in many of the kinases examined are 81.1% identical and 92.7% similar between urchins and humans (Table 2). As such, screening small molecule modulators of phosphorylation enabled the identification of candidate kinases and phosphatases that modulate attachment/priming, Ca^2+^ sensitivity and membrane fusion. One caveat of such a screening study might be that in cases in which a test compound had no effect, the target kinase or phosphatase might not be present on the vesicle membrane. However, A419259 and STO-609, inhibitors of src kinase and CaMKK, respectively, were the only compounds tested that had no effect on any of the parameters assessed (Figure 1(Aiii,Aiv,Biii,Biv)). In the case of A419259, the dual abl/src kinase inhibitor bosutinib also had no effect except for an ~3% inhibition of fusion at saturating [Ca^2+^]_free_ and only in the endpoint assay. Therefore, our results are in agreement with studies showing that tyrosine phosphorylation may modulate upstream stages of exocytosis via known proteins [19,98], but provides no evidence to suggest their involvement in the modulation of late priming/docking reactions, Ca^2+^ sensitivity or any marked effect on fusion (Figure 2A,B). It may thus be that any substantial modulatory influence of tyrosine phosphorylation may occur via target components in the plasma membrane. Similarly, although we have previously established that there is substantial calmodulin on the CV membrane [2], the data here are not consistent with a role for CaMKK except perhaps at the plasma membrane [99], although that would require verification by alternate studies.

### 4.1. Protein Kinase C, Protein Kinase A, Calmodulin-dependent Protein Kinase II 

Treatment of CV with inhibitors of PKC, PKA and CaMK II inhibited fusion in response to low [Ca^2+^]_free_, but not that triggered by a slightly higher, but still submaximal dose of [Ca^2+^]_free_ (Figure 1(Ai)). Consistent with a previous assessment of the role of CaMK I (and calmodulin) using the CV model system, the results here also showed that the EC_50_ was not altered (Figure 2A,B) [2]. Inhibition in response to low [Ca^2+^]_free_ was only observed in the endpoint assay, but not the settle assay, indicative of selective changes to Ca^2+^ sensitivity that did not result in significant changes to EC_50_. Studies of regulated exocytosis in mammalian cells have generally found similar effects following the modulation of protein kinase activity, such that higher [Ca^2+^]_free_ ‘overcome’ inhibition observed at lower [Ca^2+^]_free_, or potentiation of release following the activation of kinases is significantly more potent at low doses of [Ca^2+^]_free_ [37,100]. As mammalian cells contain functionally distinct pools of secretory vesicles (broadly defined as those belonging to slowly and rapidly releasable pools), changes in rates of secretion can either reflect changes in the sizes of particular vesicle pools or changes to the Ca^2+^ sensitivity of triggering and fusion. In the case of PKC, treatment of neuronal [100], neuroendocrine [13,37], haematopoietic [14] and β cells [12] with PMA has been shown to potentiate exocytotic release. However, PMA has also been found to bind Munc13, Rac GTPase activating proteins and guanine nucleotide-exchange factors, complicating interpretation of the release-enhancing effects. Nonetheless, overexpression of phosphomimetic mutants (i.e., at the PKC phosphorylation site) of SNAP25 and Munc18 has been found to mimic the effects of PMA [15]; treatment with specific inhibitors of PKC, such as the inhibitory peptide, have proven more selective than PMA [13,100,101]. Electrophysiological and optical studies of chromaffin cells have found an increase in the rapidly releasable pool of vesicles following activation of PKC [102], whereas studies of neuronal cells have found increases in the probability of release, without changes to pool sizes, reflective of alterations in Ca^2+^ sensitivity [100,101,103]. Here, following treatment with Gö 6983 and PKC inhibitory peptide, CV fusion triggered by low [Ca^2+^]_free_ was inhibited, but the response to medium [Ca^2+^]_free_ was not altered, and there was no significant change in Ca^2+^ sensitivity (Figure 1(Ai,Bi) and Figure 2A,B). Furthermore, PMA did not potentiate Ca^2+^ sensitivity (Figure 1(Ai) and Figure 2A,B). Furthermore, treatment of CV with 100 nM Gö 6983 and 100 nM PMA did not alter the rate of fusion triggered by ~500 µM [Ca^2+^]_free_ [35]. In neuroendocrine cells, intracellular treatment with PMA (i.e., using permeabilised cells or by perfusion via a patch pipette) caused rapid translocation of the enzyme to the plasma membrane, and treating permeabilised neuroendocrine cells with PKC inhibitors without prior exposure to PMA had no effect on Ca^2+^ sensitivity [13]. Here, CV were treated in the absence of cytosolic components, and only CV membrane-associated PKC activity (or Munc13 and other phorbol ester receptors) could have been modulated. Thus, it is likely that very low levels of PKC are associated with isolated CV. As CV fusion was not found to be significantly altered in the settle assay following treatment with PKC modulators, we find no evidence to suggest that PKC is involved in late priming/docking reactions; instead, the small, selective inhibition in response to low [Ca^2+^]_free_ in the endpoint assay likely reflects selective, albeit limited, modulation of a Ca^2+^ sensor. Nonetheless, the activities of PKA, CaMK II and CaMKK have been reported to modulate stages upstream of the late Ca^2+^-triggered steps of exocytosis [99]. It is likely that such upstream modulation was not reflected in the settle assay as CV are fully docked and release-ready, and there are no reserve pools that require additional steps to become fusion-competent; all such maturation reactions apparently occur during early oogenesis [104]. Thus, PKC, PKA, CaMK II and CamKK, along with other recently investigated enzymes [8,27], can modulate the efficiency of the subsequent fusion response as components of the physiological fusion machinery and could have selective influences on a Ca^2+^ sensor [1,2,8,9]. Studies linking PKC activity to the modulation of fusion pore expansion [14] are consistent with this interpretation. As the assays utilised here do not assess fusion pore expansion per se, we cannot comment further on potential modulation via such a mechanism.

### 4.2. Rho Kinase

Rho kinase (ROCK activity is modulated by the Rho family of small GTPases, and ROCK is known to phosphorylate proteins involved in exocytosis (e.g., syntaxin-1 [105]). Several studies have reported an inhibition of exocytotic release following activation of ROCK. Treatment of primary pancreatic β-cells with ROCK inhibitors potentiated insulin release apparently by accelerating the depolymerisation of actin and thus likely facilitating vesicle–plasma membrane contact [106], and activation of the catalytic domain of ROCK inhibited release of human growth hormone from PC12 cells (with this effect also seen following overexpression of constitutively active mutants of Rho GTPases) [107]. Treatment of rat brain cortical synaptosomes with a ROCK inhibitor, Y27632, potentiated KCl-triggered exocytosis, whereas a myosin phosphatase inhibitor inhibited exocytosis. In this study, GSK 429286, a highly selective ROCK inhibitor, was shown to selectively potentiate CV fusion in the settle assay (Figure 1(Bii)), implicating the kinase as a negative regulatory component of priming/docking and, thus, a modulator of the late steps of exocytosis.

### 4.3. Casein Kinase 2

Casein kinase 2 (CK2) is known to phosphorylate several proteins involved in regulated exocytosis, including syntaxin 1-A [108] and synaptobrevin [109]. In vitro, phosphorylation of syntaxin 1A by CK2 enhances its binding to the C2B Ca^2+^ binding domain of synaptotagmin 1 and its interaction with Munc18 [108], and phosphorylation of complexin by CK2 enhances its binding to the SNARE complex [110]. Treatment of rat brain synaptosomes with 25 µM 2-dimethylamino-4,5,6,7-tetrabromo-1*H*-benzimidazole (DMAT) resulted in potentiation of the rate of glutamate release, and this change correlated with a reduction of syntaxin 1A phosphorylation in detergent-resistant membranes [111]. Here, the same dose of TBB (a new class of CK2 inhibitor derived from DMAT) selectively potentiated the EC_50_ for Ca^2+^ in the endpoint assay (Figure 3A), implicating CK2 in the modulation of Ca^2+^ sensitivity. Previous studies have implicated SNARE proteins in promoting or maintaining the Ca^2+^ sensitivity of fusion in a physiologically-relevant range [24,25,26], and phosphorylation mediated protein–protein interactions are clearly implicated in facilitating SNARE interactions that contribute to vesicle docking/priming and thus subsequent efficient release [112].

### 4.4. Dephosphorylation

The data here indicate that multiple phosphatases are present and active on the CV membrane and thus represent fundamental components of the late steps of triggered release. CV fusion was potentiated in the endpoint assay by α-NP and CaN peptide, whereas okadaic acid selectively potentiated fusion in the settle assay and had no effect in the endpoint assay (Figure 1(Aii,Bii) and Figure 3A,B), in agreement with a previous study showing that okadaic acid had no effect on CV–plasma membrane fusion [28]. As okadaic acid targets protein phosphatases 1 and 2A, this suggests their involvement in the modulation of priming/docking. Furthermore, α-NP also had a pronounced effect on Ca^2+^ sensitivity, significantly lowering the EC_50_ for fusion (Figure 3A,B), and the activity of CaN was also implicated in the modulation of Ca^2+^ sensitivity (Figure 1(Av,Bv)). As neither okadaic acid nor CaN peptide mimicked the effects of α-NP, it is likely that other phosphatases present on the CV membrane further influence the late Ca^2+^-triggered steps of regulated exocytosis. Furthermore, as the leftward shift in EC_50_ following treatment with α-NP was comparable in the endpoint and settle assays (Figure 3A,B), we cannot directly discriminate between effects on Ca^2+^ sensitivity versus those on priming and docking. Nonetheless, previous studies have found more pronounced effects in the settle assay when priming components are targeted [8,9,27], and it thus seems likely that treatment with α-NP does influence Ca^2+^ sensitivity, perhaps of the docking, priming and fusion steps; indeed, the inhibitory effects of α-NP indicate a potentiating role on fusion pore expansion for an as yet unknown phosphatase [14]. In addition to potentiating effects, α-NP also caused minor inhibition of the extent of fusion, further suggesting the presence of multiple phosphatases that play diverse roles to regulate the late stages of exocytosis. Overall, our data indicate that inhibiting phosphatase activity potentiates release, and this is in agreement with findings in numerous cell types, though further work is required to identify the specific interactions that modulate Ca^2+^ sensitivity [12].

### 4.5. Lipid Phosphorylation

Phosphorylation of lipids can generate bioactive secondary metabolites that are capable of modulating diverse cellular processes. Phosphorylation of diacylglycerol by diacylglycerol (DAG) kinase generates phosphatidic acid (PA), an anionic lipid that has been associated with docking and priming roles, likely as a site for selective binding of different protein components [27]. In contrast, diacylglycerol has been extensively investigated in relation to its activation of PKC [113] and potential role in facilitating the initial step of membrane merger by virtue of its pronounced intrinsic negative curvature [7,10]. Indeed, studies of sea urchin embryos have shown that diacylglycerol modulates the fusion of precursor vesicles required for the formation of the nuclear envelope during mitosis [114]. Here, treatment of CV with the DAG kinase inhibitor R59022 selectively inhibited fusion in response to low [Ca^2+^]_free_, but only in the endpoint assay (Figure 1(Avi)); thus, as with PKC and other kinases described above, DAG kinase activity likely has a selective, but limited influence on a Ca^2+^ sensor.

Several recent studies have found that changes in sphingolipid levels may influence distinct stages of the regulated exocytotic pathway, either via activation of downstream signalling mechanisms that activate enzymes such as phospholipase C or by accelerating the assembly of SNARE complexes [115,116,117,118,119,120,121]. Inhibition of sphingosine and ceramide phosphorylation leads to increased levels of these substrates and decreased levels of sphingosine-1-phosphate and ceramide-1-phosphate, respectively. Although sphingolipid biophysical properties have been shown to influence the overall curvature and permeability of model cell membranes [122], their influence on membrane merger has not been directly examined in native regulated exocytotic systems. Here, treatment of CV with NVP231, a ceramide kinase inhibitor, caused a slight potentiation in the settle fusion assay (Figure 1(Bvi)), whereas treatment with DMS, a sphingosine kinase inhibitor, potently inhibited CV fusion even in response to a saturating [Ca^2+^]_free_ (Figure 1(Avi,Bvi)). Indeed, the latter was the most significant effect of any of the compounds tested. As such, regulation of sphingolipid levels by ceramide and sphingosine kinase is implicated in modulating docking/priming and membrane fusion, respectively. Thus, while ceramide kinase may be a component of the PFM, sphingosine kinase is implicated as a likely component of the FFM, producing a localized supply of sphingosine-1-phosphate.

## 5. Conclusions

The study of CV fusion has enabled targeted identification of components of the conserved molecular mechanism underlying the late Ca^2+^-triggered steps of regulated exocytosis, and here, new high-throughput assay formats have identified candidate kinases and phosphatases, on the secretory vesicle membrane, that modulate priming/docking, Ca^2+^ sensitivity and membrane fusion. The new assays formats used in this study are cost-effective, provide direct access to the exocytotic mechanism(s) and do not involve cell isolation from vertebrate, mammalian sources. The results indicate the presence of multiple phosphorylation sites on proteins and lipids, localised on the secretory vesicle membrane, serving as components of both the PFM and FFM, and this is consistent with a recent analysis of protein phosphosites in synaptosomes [123]. Uniquely, the approach here also identified the critical importance of lipid phosphorylation, in particular of sphingosine. Specifically, phosphatases and CK2 are implicated in modulating Ca^2+^ sensitivity, and sphingosine kinase activity is implicated more directly in enabling membrane fusion. While it is also possible that kinases and phosphatases in the local cytosol might also modulate components of the PFM and FFM, here we sought only to assess the effects of vesicle-associated enzymes. In any case, it would be difficult to assess local cytosol effects or components specifically near the site of docking and fusion. This study thus defines a modified, high-throughput fusion assay format to better capitalize on the well-established CV model system. The results provide a rationale for larger scale proteomic and lipidomic analyses of CV membranes to identify the CK2 and phosphatase substrates and the potentially wider lipid alterations resulting from sphingosine kinase inhibition, in order to further identify critical components and correlate molecular changes with alterations in specific steps underling triggered exocytotic release. The data also provide a rationale for examining the role of CK2 and sphingosine kinase in the modulation of regulated exocytosis in mammalian cells, using techniques including high temporal resolution patch clamp assays of membrane capacitance and fusion pore expansion and a range of imaging techniques (e.g., total internal reflection fluorescence (TIRF) and stimulated emission depletion (STED) microscopy).

## Figures and Tables

**Table 1:** List of compounds utilised in this study, their molecular targets and references from which the effective dose was identified.

Compound	Molecular Target	Dose Used	Refs
Phorbol 12-myristate 13-acetate (PMA)	PKC activator	100 nM	[12,14,37,38,39,40]
Gö 6983	PKC inhibitor	10 µM	[41,42,43,44,45,46]
PKC inhibitory peptide (19–36; RFARKGALRQKNVHEVKN)	PKC inhibitor	10 µM	[13,47,48]
Staurosporine	Broad spectrum kinase inhibitor	4 µM	[49,50,51,52,53,54,55,56,57,58]
α-naphthyl phosphate (α-NP)	Broad spectrum phosphatase inhibitor	5 mM	[14]
Okadaic acid	Dual protein phosphatase 1/2A inhibitor	1 µM	[12,20,28,59,60,61,62]
Calcineurin (CaN) inhibitory peptide (457–482; ITSFEEAKGLDRINERMPPRRDAMP)	CaN inhibitor	100 µM	[20,63]
PKA inhibitory peptide (6–22; TYADFIASGRTGRRNAI)	Protein kinase A inhibitor	1 µM	[64,65,66]
4,5,6,7-tetrabromobenzotriazole (TBB)	Casein kinase 2 (Ck2) inhibitor	25 µM	[67,68]
GSK 429286	Rho kinase (ROCK) inhibitor	2 µM	[69,70,71,72]
Bosutinib	Dual abl/src kinase inhibitor	10 µM	[73,74,75,76]
A419259	Src kinase inhibitor	500 nM	[77,78,79,80,81]
Autocamtide-2-related inhibitory peptide (AIP) (KKALRRQEAVDAL)	Calmodulin-dependent protein kinase II (CaMK II) inhibitor	10 µM	[82,83]
STO609	Ca2^+^/calmodulin-dependent protein kinase kinase (CaMK kinase) inhibitor	25 µM	[84,85,86,87]
R59022	Diacylglycerol kinase inhibitor	12.5 µM	[88,89]
NVP231	Ceramide kinase inhibitor	100 nM	[90,91,92,93]
Dimethylsphingosine (DMS)	Sphingosine kinase inhibitor	100 µM	[94]

PKC: Protein kinase C; PKA: Protein kinase A.

**Table 2:** Conservation of kinase and phosphatase amino acid sequences from sea urchin to human.

Enzyme Name	Percent Identity	Percent Similarity
Protein kinase C	68.4	80
PKC conserved region 1 (phorbol ester binding site)	90	92
Serine/threonine catalytic domain	81.1	92.7
cAMP-dependent protein kinase catalytic subunit αisoform	78.7	89.8
Casein kinase 2 α subunit	79.1	86.9
Abl-related protein tyrosine kinase	69.1	80.6
Src-family protein tyrosine kinase	54.4	67.9
Rho kinase	47.2	64.4
Rho kinase associated coiled coil (containing PH domain)	55.4	69.6
Calmodulin-dependent protein kinase 1	57.6	76.6
Calmodulin-dependent protein kinase kinase	33.4	44.3
Protein phosphatase 2A catalytic subunit	94.4	97.4
Calcineurin B homologous protein	62.2	74.0
Diacylglycerol kinase	52.8	65.8
Ceramide kinase	44.8	66.5
Sphingosine kinase	43.3	61.0
Sphingosine kinase conserved domains:		
C 1	64.7	76.5
C 2	40.0	72.0
C 3	75.0	91.7
C 4	57.1	82.1
C 5	80.0	100.0
